# “Water-in-Salt” Electrolyte Suppressed MnVOPO_4_·2H_2_O Cathode Dissolution for Stable High-Voltage Platform and Cycling Performance for Aqueous Zinc Metal Battery

**DOI:** 10.3390/ma17184456

**Published:** 2024-09-11

**Authors:** Shaohua Zhu, Wenwei Zhang, Xiaobin Liao, Lei Zhang, Qinyou An, Xuanpeng Wang

**Affiliations:** State Key Laboratory of Advanced Technology for Materials Synthesis and Processing, Wuhan University of Technology, Wuhan 430070, China; zhushaohua@whut.edu.cn (S.Z.); zhangww2022@whut.edu.cn (W.Z.); liaoxiaobin@live.com (X.L.); zhanglei1990@whut.edu.cn (L.Z.); anqinyou86@whut.edu.cn (Q.A.)

**Keywords:** aqueous zinc metal battery, water-in-salt electrolyte, MnVOPO_4_·2H_2_O, vanadium dissolution, high voltage

## Abstract

Vanadium-based materials have the advantages of abundant valence states and stable structures, having great application potential as cathode materials in metal-ion batteries. However, their low voltage and vanadium dissolution in traditional water-based electrolytes greatly limit their application and development in aqueous zinc metal batteries (AZMBs). Herein, phosphate- and vanadium-based cathode materials (MnVOPO_4_·2H_2_O) with stacked layers and few defects were prepared via a condensation reflux method and then combined with a high-concentration electrolyte (21 m LiTFSI + 1 M Zn(CF_3_SO_3_)_2_) to address these limitations. The specific capacity and cycle stability accompanying the stable high voltage of 1.39 V were significantly enhanced compared with those for the traditional electrolyte of 3 M Zn(CF_3_SO_3_)_2_, benefiting from the suppressed vanadium dissolution. The cathode materials of MnVOPO_4_·2H_2_O achieved a high specific capacity of 152 mAh g^−1^ at 0.2 A g^−1^, with a retention rate of 86% after 100 cycles for AZMBs. A high energy density of 211.78 Wh kg^−1^ was also achieved. This strategy could illuminate the significance of electrolyte modification and provide potential high-voltage cathode materials for AZMBs and other rechargeable batteries.

## 1. Introduction

Rechargeable aqueous zinc metal batteries (AZMBs) are considered one of the most promising next-generation energy storage systems due to their low cost, environmental friendliness, and high theoretical specific capacity, along with the low redox potential of zinc (−0.76 V vs. the standard hydrogen electrode (SHE)) [1,2]. Among the varied cathode materials in AZMBs, such as vanadium, manganese, and Prussian blue cathodes, vanadium-based materials have received more attention due to their multiple valence states and suitable structures, which usually exhibit a high specific capacity, good magnification performance, and long cycle life [3,4,5]. Typically, V_2_O_5_ [6], V_6_O_13_ [7], and other vanadate-based cathodes [8] have demonstrated excellent performance, including a high capacity and stable cycling life. However, the further application of vanadium cathodes remains plagued by the following issues.

On the one hand, the average operating voltage of vanadium oxides tends to be very low (approximately 0.8 V), which severely limits their energy density. Polyanionic electrode materials with high charging and discharging voltage platforms have been widely studied in lithium-ion batteries to achieve a high energy density. Thus, combining a high-capacity vanadium compound with a high-voltage polyanion group to develop new cathode materials is expected to achieve a high energy density and stable cycling life. Chen et al. recently focused on applying vanadium oxide phosphate with different phase structures in AZMBs. As a cathode material, δ-VOPO_4_ has a higher operating voltage of 1.46 V and excellent cycle stability [9]. Moreover, other types of VOPO_4_ and other polyanion-based cathodes have been developed for high-voltage plateaus to achieve high energy densities [10,11,12,13].

On the other hand, the high reactivity of water in a traditional electrolyte causes the corrosion of the metal zinc, hydrogen evolution, and other side reactions, as well as the dissolution of the cathode material, typically a vanadium-based material, causing the battery’s capacity to rapidly decay and fail [14,15]. Water decomposition occurs especially when operating with higher and wider voltage windows. Therefore, reducing the suitably free water may be an effective modification strategy. Introducing appropriate additives to regulate active water through hydrogen bonding is a widely used strategy [16,17,18]. Although the modification effect is excellent, a small amount of additives may not be able to improve these problems. Comparatively, using a salt-coated water electrolyte is a typical strategy to reduce free water in which many anions occupy the solvation structure and the cathode surface, avoiding direct contact between the water and the cathode material to ease the dissolution of active elements [19]. Wang et al. [20] prepared an ultra-low-water-activity electrolyte by introducing the internal diluent TMP, which significantly suppressed the negative reaction and the vanadium dissolved in the solution. Furthermore, V_6_O_13_ showed excellent stability with a capacity retention rate of 99.43% after 3000 cycles at 1 A g^−1^ and set a new record for 30,000 cycles in a zinc-ion battery. This strategy also received more attention in AZMBs and was successfully applied in other metal-ion batteries with outstanding modification results [21,22,23].

Thus, we can solve the challenges faced by AZMBs, especially high-voltage vanadium-based cathodes, by combining the above two strategies.

Here, we prepared a novel MnVOPO_4_·*n*H_2_O cathode material and constructed a highly stable MnVOPO_4_·*n*H_2_O//21 m LiTFSI + 1 M Zn(CF_3_SO_3_)_2_//Zn battery system. The material immersion experiment proved that it can inhibit the dissolution of the cathode material. The modification effect was further verified via electrochemical performance evaluation. Finally, using XPS, ex situ XRD, optical observations, etc., we found that the oxidation and reduction of V are highly reversible, indicating that our strategy has a significant inhibitory effect on vanadium dissolution. This work provides new ideas for developing high-voltage, high-stability, and high-capacity AZIB systems.

## 2. Materials and Methods

### 2.1. Synthesis of Materials

All chemicals were used directly after purchase without any pretreatment.

MnVOPO_4_·2H_2_O was prepared by condensation reflux. Briefly, 2.4 g of V_2_O_5_ (Macklin, AR, Shanghai, China, ≥99.5%) and 0.82 g of KMnO_4_ (Macklin, 99.99%) were added to a beaker. Then, 16 mL of H_3_PO_4_ (mass fraction 85%, Macklin) and 55 mL of deionized water were added to the beaker, which was placed in a magnetic mixer and stirred at room temperature for 5 min. At this point, the solution mixture appeared wine-red. After that, the solution mixture in the beaker was transferred to a round-bottom flask, in which a stir bar was placed for stirring. The round-bottom flask was placed in a constant-temperature magnetic-stirring oil bath set at 130 °C for 16 h. The mixture solution in the round-bottom flask was filtered after the reaction. The brown solid substance thus obtained was cleaned with deionized water and then dried in a vacuum-drying oven at 70 °C.

### 2.2. Preparation of Electrolyte

#### 2.2.1. High-Concentration Electrolyte

An amount of 21 m of LiTFSI was prepared with 1 M of Zn(CF_3_SO_3_)_2_ (TCI, >99.0% (T)). The solvent was deionized water. The specific preparation process was to weigh the required LiTFSI in a lithium battery glove box to avoid the influence of electrolyte moisture absorption deterioration, quickly add Zn(CF_3_SO_3_)_2_ and deionized water in the air atmosphere, and then cover the bottle with a sealing film to seal the container. The mixture was placed in a magnetic mixer and stirred for more than 24 h until the solution was completely clear. This electrolyte should be used up within three days after its preparation to avoid deterioration due to moisture absorption.

#### 2.2.2. Phosphate Buffer-Type Electrolyte Additives

An amount of 3 M of Zn(CF_3_SO_3_)_2_ was prepared with phosphate buffers at different concentrations (0.004 M, 0.1 M). The phosphate buffers comprised Na_2_HPO_4_ and NaH_2_PO_4_ at the same concentration. The solvent was deionized water. The specific preparation process was, first, to add Na_2_HPO_4_ (Macklin, 99.99%) and NaH_2_PO_4_ powder (Macklin, 99.99%) as phosphate buffers, then deionized water, and, finally, Zn(CF_3_SO_3_)_2_ powder to avoid the appearance of insoluble matter.

#### 2.2.3. MnSO_4_ Electrolyte Additive

An amount of 3 M of Zn(CF_3_SO_3_)_2_ was prepared with different concentrations of MnSO_4_ (0.2 M and 0.5 M, Macklin, 99.99%). The solvent was deionized water. The specific preparation process was, first, to add MnSO_4_ powder, then deionized water, and, finally, Zn(CF_3_SO_3_)_2_ powder.

### 2.3. Material Characterization

The crystallographic characterization of the cathode materials was performed with a Bruker D8 Discover XRD device with Cu Kα radiation (λ = 1.5418 Å). SEM images were collected with a JEOL-7100F microscope with an acceleration voltage of 20 kV. TEM images, energy-dispersive X-ray spectroscopy (EDX) element mappings, and high-resolution transmission electron microscopy (HRTEM) images were recorded using a Titan G2 60–300 transmission electron microscope. Raman spectrum measurements were implemented with a Renishaw RM-1000 laser Raman microscopy system. X-ray photoelectron spectroscopy (XPS) measurements were achieved using a VG K-Alpha Probe spectrometer (Thermofisher Scientific, Waltham, MA, USA) with Al Ka radiation as the excitation source. Raman (Renishaw INVIA, New Orleans, LA, USA) was applied to obtain the spectra of the cathode material.

### 2.4. Electrochemical Measurements

The cathode was prepared via the traditional process of mixing MnVOPO_4_·2H_2_O, PVDF, and acetylene black in a ratio of 6:3:1, forming a uniform mixed slurry. Then, the slurry was uniformly coated on titanium foil and handled in a drying box at 60 °C for 12 h. Finally, the slurry-coated titanium foil was punched into an electrode sheet with a diameter of 10 mm.

Subsequently, CR-2016-type coin cells were assembled in air using the prepared electrolyte, zinc foil (100 μm), and glass fiber membranes (Whatman GF-D) as the electrolyte, anode, and separator, respectively. Then, the LAND battery testing system (CT2001A, Wuhan, China) was used to evaluate the electrochemical performance. The cyclic voltammetry (CV, 0.8–2.1 V) and electrochemical impedance spectroscopy (EIS) measurements (0.01−1 × 10^5^ Hz) were made using an electrochemical workstation (CHI760E) at room temperature.

### 2.5. Operation before Ex Situ XRD and XPS Analysis

Electrode sheets for the ex situ characterizations (XRD and XPS) were obtained from the batteries operating at a current density of 0.1 A g^−^^1^ over a voltage range of 0.8–2.1 V during the second discharge/charge cycle. Then, these electrodes were washed several times with absolute ethanol and dried in a glove box at room temperature for 24 h.

## 3. Results and Discussion

**Figure 1:**
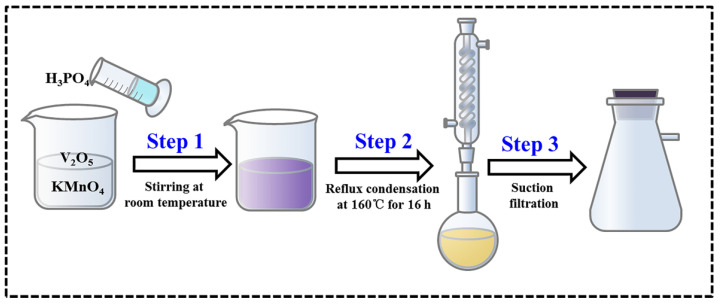
Synthetic process schematic of MnVOPO_4_·2H_2_O.

As shown in Figure 1, MnVOPO_4_·2H_2_O was prepared using the condensation reflux method at room temperature. Specifically, V_2_O_5_ and KMnO_4_ were added to a beaker. Then, H_3_PO_4_ and deionized water were added to the beaker, and the beaker was placed on a magnetic mixer and stirred at room temperature. At this point, the solution mixture appeared wine-red. After that, the solution mixture in the beaker was transferred to a round-bottom flask, in which a stir bar was placed for stirring. The round-bottom flask was placed in a constant-temperature magnetic-stirring oil bath set at 130 °C for 16 h.

**Figure 2:**
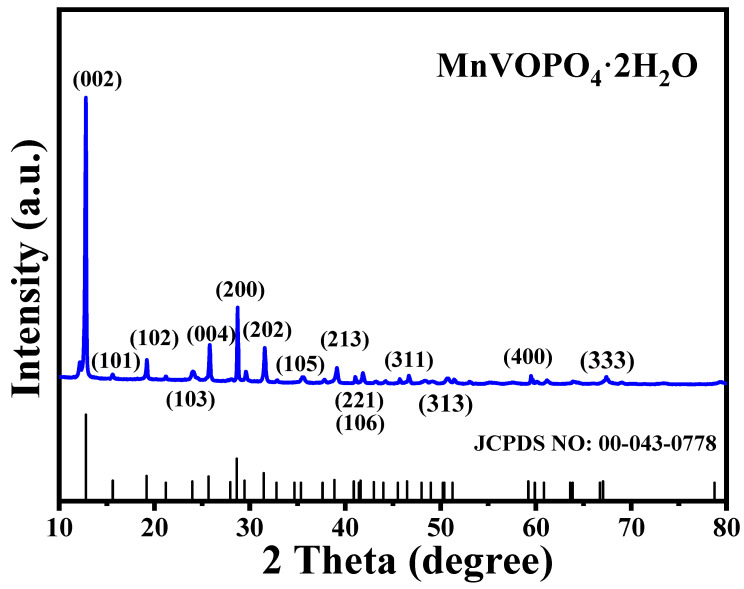
The XRD pattern of MnVOPO_4_·2H_2_O.

The X-ray diffraction pattern of MnVOPO_4_·2H_2_O matches with the JCPDS: 00-043-0778 card (a = 6.2034, b = 6.2034, c = 13.8140, and α = β = γ = 90°), which demonstrates its high purity (Figure 2). In addition, it shows preferential growth along the (002) crystal surface, which accords with the growth law of 2D layer materials and the existing literature [24,25]. Moreover, the average grain size of the main diffraction peaks is distributed in the range of 50–60 nm (Table 1). In general, this small grain size can reduce the diffusion path of zinc ions and promote the mass transfer process.

Moreover, in the XRD pattern, the position and intensity of the diffraction peaks reflect information about the periodicity of the crystal structure and the arrangement of atoms. Defects in the material, such as dislocations, vacancies, or impurities, can change the arrangement of the atoms, which affects the position and strength of the diffraction peaks. Firstly, the interplanar distance is increased, corresponding to the smaller 2 theta value of the experimental data than that of the standard PDF card according to the Bragg equation (Table 1). This is due to the lattice change due to the stress caused by the infiltration of other atoms into the lattice. In addition, the strength ratio of 0.259 between the (200) and (002) planes shows a downward trend compared with that of the standard database (0.36), indicating changes in the local chemical structure of the material and proving that there are defects in the material.

**Figure 3:**
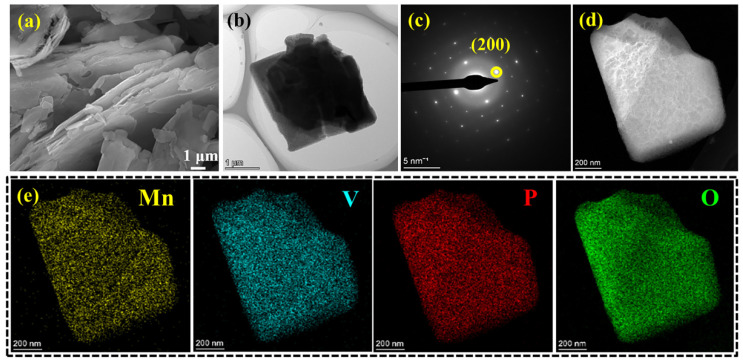
(**a**,**b**) SEM and TEM images, (**c**) SAED, and (**d**,**e**) mapping images of MnVOPO_4_·2H_2_O sample.

Moreover, the SEM image demonstrates that MnVOPO_4_·2H_2_O shows a layered stacking structure at the micron scale, with a blocky distribution of materials. Each piece is stacked in layers with an approximate size of 2 × 2 × 0.1 μm (Figure 3a,b). Furthermore, it shows a layered stacking profile structure, and each layer has the same width, showing a certain rule. This block-distributed layered morphology may be conducive to the penetration of the electrolyte and promote the diffusion kinetics of zinc ions. In addition, the pattern of electron diffraction in selected regions was characterized by selected area electron diffraction (SAED) (Figure 3c). The electron diffraction patterns show that MnVOPO_4_·2H_2_O has the properties of a single crystal and has a tetragonal structure. The diffraction pattern calibration of the (200) crystal surface is consistent with the XRD results, which plays a complementary role in the high purity of the synthesized MnVOPO_4_·2H_2_O [24,25,26]. The TEM results further demonstrate this structure. The manganese, vanadium, phosphorus, and oxygen are uniformly distributed (Figure 3d,e).

**Figure 4:**
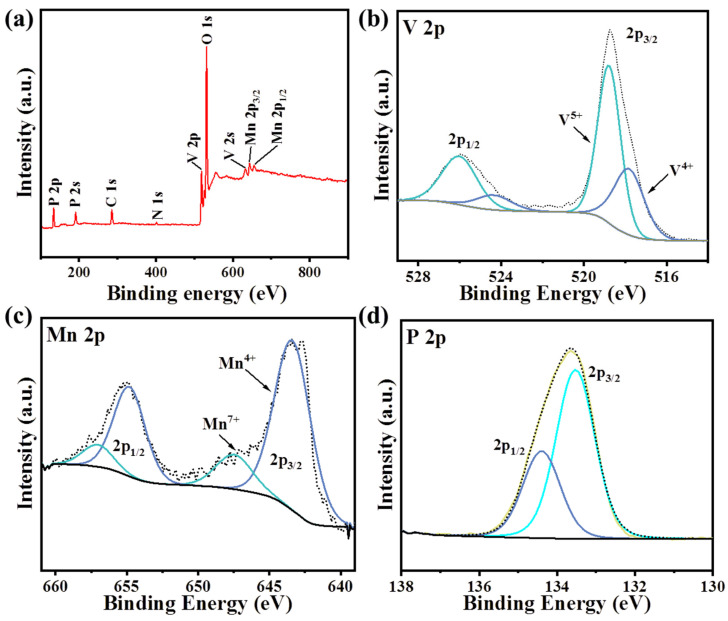
(**a**–**d**) Full, V 2p, Mn 2p, and P 2p XPS spectra of MnVOPO_4_·2H_2_O sample.

An XPS full-spectrum analysis of MnVOPO_4_·2H_2_O was carried out, in which the target was Al Kα. The full spectrum was corrected during the data processing using the polluting carbon binding energy of 284.8 eV. The test results show that the synthesized sample contained only four elements, V, P, O, and Mn, which demonstrated the high purity of the synthesized sample (Figure 4a). In Figure 4b, the V 2p 3/2 and V 2p 1/2 peaks of the sample confirm that the vanadium element was in the mixed-valence state (V^4+^/V^5+^), which corresponds to the V-O bond [10,11]. Moreover, this mixed state may help obtain higher electrochemical activity, lower polarization, faster ion diffusion, and higher electrical conductivity for MnVOPO_4_·2H_2_O [27]. Figure 4c also shows a spin–orbit splitting in the Mn 2p region for manganese. The difference between the peak corresponding to the 2p 1/2 orbit and the peak corresponding to the 2p 3/2 orbit is 11.2 eV. Based on this, two groups of peaks can be obtained via sub-peak fitting. When the binding energy of the Mn 2p 3/2 orbital corresponds to 642.8 eV, it corresponds to Mn-^3+^ (Mn-O), which is consistent with the XRD results. When this value is 647 eV, it corresponds to the highest oxidation state of manganese, heptavalent manganese, because one of the synthetic byproducts of MnVOPO_4_·2H_2_O is HMnO_4_. The appearance of this peak comes from a small amount of HMnO_4_ residue on the sample surface rather than the unfinished KMnO_4_. Figure 4d also shows a spin–orbit split in the 2p region, where the difference between the peak corresponding to the 2p 1/2 orbit and the peak corresponding to the 2p 3/2 orbit is 1 eV. Moreover, the fitting peak corresponding to the P 2p 3/2 is 133.5 eV, which is the binding energy of phosphorus in a typical phosphate group.

**Figure 5:**
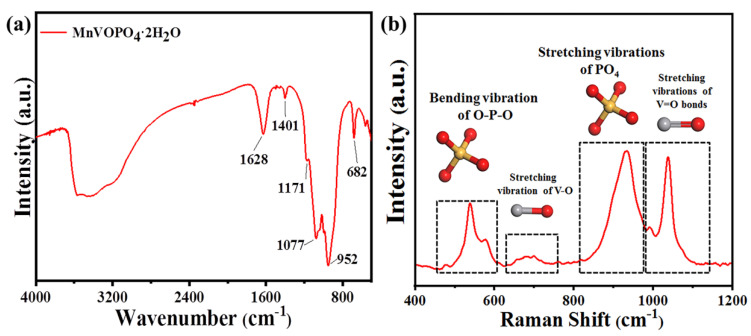
(**a**,**b**) FT-IR and Raman spectrogram of MnVOPO_4_·2H_2_O sample.

As shown in the FT-IR spectrum in Figure 5a, the transmission peaks at 1628 cm^−1^ and 1611 cm^−1^ could correspond to the bending vibration of H-O-H in water molecules. Furthermore, the higher wave number and weak vibration are related to the weakened hydrogen bond between the water molecules and phosphate groups. It can be speculated that the embedding of manganese causes some water molecules to bond with manganese to form MnO5 (H_2_O) octahedrons, thus reducing the number of water molecules to form hydrogen bonds with phosphate groups. The peaks at 1171 cm^−1^ and 1167 cm^−1^ correspond to the stretching vibration of P-O in PO_4_^3−^, demonstrating the presence of a phosphate group. The peak at 682 cm^−1^ corresponds to the bending vibration of V-O-P, proving that the vanadium ion is connected to O in PO_4_^3−^ [9].

Moreover, according to the Raman spectrum results in Figure 5b, the peak at 933 cm^−1^ in MnVOPO_4_·2H_2_O corresponds to the symmetric stretching vibration of O-P-O, while the peaks at 538 cm^−1^ and 576 cm^−1^ correspond to the bending vibration of O-P-O. In addition, the peak at 692 cm^−1^ corresponds to the stretching vibration of V-O, and the peaks at 992 cm^−1^ and 1035 cm^−1^ correspond to the stretching vibration of V=O. These results are consistent with the FT-IR and other results [9,24].

Hence, high-purity MnVOPO_4_·2H_2_O was successfully prepared.

**Figure 6:**
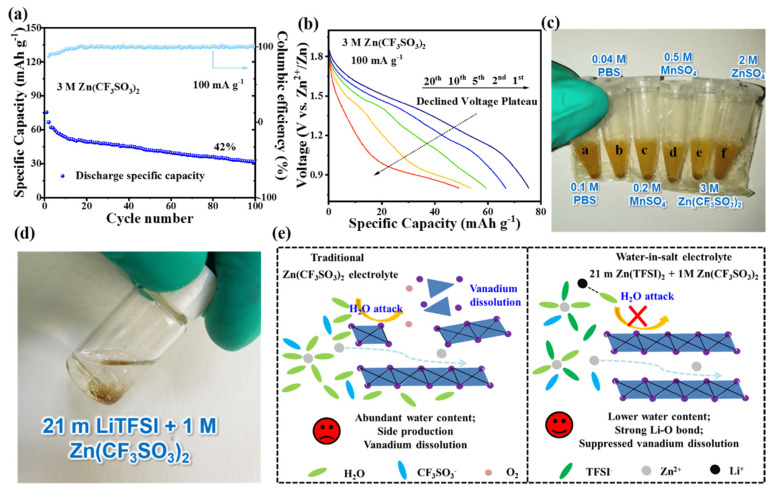
(**a**,**b**) The cycling performance and corresponding discharge curves at 0.1 A g^−1^. (**c**,**d**) Optical photographs of dissolution of MnVOPO_4_·2H_2_O in 3 M Zn(CF_3_SO_3_)_2_ and other additive-modified electrolytes after one week, as well as 21 m LiTFSI + 1 M Zn(CF_3_SO_3_)_2_ after two months. (**e**) Schematic illustrations of vanadium dissolution in traditional and “water-in-salt” electrolytes.

Subsequently, as shown in Figure 6a, the MnVOPO_4_·2H_2_O//3 M Zn(CF_3_SO_3_)_2_//Zn battery was assembled, but it had poor cycle stability and low specific capacity. Specifically, the battery’s maximum discharge specific capacity was only 75 mAh g^−1^ at 0.1 A g^−1^ within the voltage range of 0.8 to 1.9 V, and the capacity retention rate was only 42% after 100 cycles. The discharge medium voltage at the beginning of the cycle reached 1.36 V, which is much higher than the average 0.45–0.85 V of vanadium-based cathode materials used in AZMBs, indicating its potential for high-voltage working platforms (Figure 6b) [28,29,30]. However, the discharge medium voltage gradually decreased to 0.94 V within the first 20 charge and discharge cycles, and the Coulombic efficiency was less than 100% in the first 20 cycles. It can be inferred that the cathode material suffered serious structural damage at the early stage of the electrochemical reaction, which hindered the ability of MnVOPO_4_·2H_2_O to maintain a high-voltage platform for zinc storage.

It was expected that the above issues could be alleviated via the common different modification strategy by preparing other electrolytes based on the electrolyte bulk property. Obvious dissolution appeared after one week when MnVOPO_4_·2H_2_O was immersed in various electrolytes based on a regulated chemical potential, 3M Zn(CF_3_SO_3_)_2_, and a common 2 M ZnSO_4_ electrolyte for AZMBs. Meanwhile, the high-concentration electrolyte 21 m LiTFSI + 1 M Zn(CF_3_SO_3_)_2_ combination showed no obvious dissolution after soaking for up to two months, which initially demonstrated the excellent ability of the high-concentration electrolyte to inhibit material dissolution (Figure 6c,d). Usually, there are more H_2_O and CF_3_SO_3_^−^ anion groups around the Zn^2+^ ions in traditional electrolytes, and the active H_2_O is located at the cathode/electrolyte interface after desolvation (Figure 6e). Moreover, the active H_2_O attacks the cathode to cause vanadium dissolution and other side reactions. As a result, the poor electrochemical performance is mainly caused by serious vanadium dissolution (not the Mn element and PO_4_^3−^ group) in MnVOPO_4_·2H_2_O due to the abundant H_2_O content in traditional electrolytes (Figure 6e). A strong bond forms between Li^+^ and the oxygen atoms in water molecules to inhibit the decomposition of water in “water-in-salt” electrolytes to exceed the high-voltage range of 1.98 V [31]. Furthermore, more anion groups participate in the Zn^2+^ solvation structure and decrease the active H_2_O at the cathode/electrolyte interface to suppress vanadium dissolution. Thus, the “water-in-salt” electrolyte including 21 m LiTFSI + 1 M Zn(CF_3_SO_3_)_2_ was selected as a promising electrolyte for subsequent electrochemical performance in the current research.

Then, the Zn^2+^ storage behavior of MnVOPO_4_·2H_2_O was evaluated using CR2016-type coin cells in the selected 21 m LiTFSI + 1 M Zn(CF_3_SO_3_)_2_ electrolyte.

**Figure 7:**
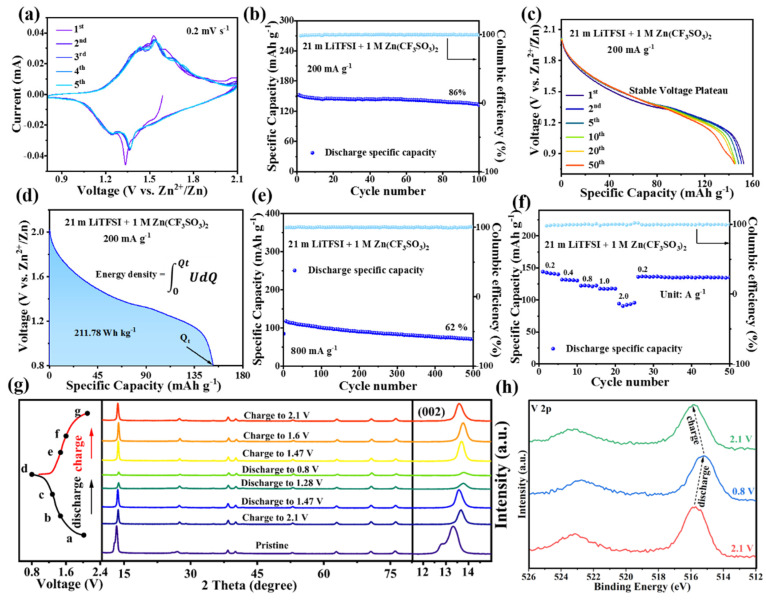
(**a**) CV curves in the first 5 cycles. (**b**,**c**) Cycling performance and corresponding discharge curves at 0.2 A g^−1^. (**d**) Energy density and 1st discharge curve. (**e**) Cycling performance at 0.8 A g^−1^. (**f**) Rate performance at different current densities. (**g**) The ex situ XRD pattern during the 2nd cycling process and the detailed change in the typical (002) plane. The letters a–g represent charge to 2.1 V, discharge to 1.47 V, discharge to 1.28 V, discharge to 0.8 V, charge to 1.47 V, charge to 1.6 V, and charge to 2.1 V, respectively. (**h**) XPS results for V 2p in fully discharged and charged states.

Firstly, two pairs of redox peaks appeared at 0.2 mV s^−1^ within the voltage range of 0.8–2.1 V, both of which were vanadium redox peaks (1.54/1.37 V and 1.44/1.25 V) (Figure 7a). Meanwhile, the highly reversible CV curves predicted considerable cycling reversibility with high-concentration electrolytes. Then, the specific discharge capacity of the battery reached 152 mAh g^−1^ at 200 mA g^−1^, and the capacity retention rate reached 86% after 100 cycles at this low current density. Moreover, the discharge curve had a high degree of coincidence, even after 50 cycles, which was consistent with the changes in the CV curve (Figure 7c). Furthermore, an energy density of 211.78 Wh kg^−1^ and discharge voltage platform of 1.39 V were reached based on the following calculations (Figure 7d).

(1) $$E=\int_{0}^{Qt}UdQ$$

(2) $$U_{\mathrm{ave}}=\frac{1}{Qt}\int_{0}^{Qt}UdQ$$

The specific capacity and cycle stability of the battery were more than doubled compared with those of the battery without electrolyte control. Significantly, the high discharge voltage was steadily retained in this “water-in-salt” electrolyte. It also exhibited a higher voltage platform than the common vanadium and organic-based cathodes [28,29,30,32,33,34] and even other polyanionic cathode materials (Table 2) [35,36].

Subsequently, the specific discharge capacity reached 118 mAh g^−1^ when the current density was increased to 800 mA g^−1^. The specific discharge capacity was maintained at 71 mAh g^−1^ after 500 cycles (Figure 7e). Moreover, the Zn/MnVOPO_4_·2H_2_O batteries based on the 21 m LiTFSI + 1 M Zn(CF_3_SO_3_)_2_ electrolyte showed excellent rate performance (Figure 7f). They maintained a specific discharge capacity of 95 mAh g^−1^ when the current density was increased to a high value of 2 A g^−1^. Significantly, the specific capacity almost returned to the initial value and maintained a stable cycle as the current density decreased from 2A g^−1^ to 0.2 A g^−1^. Thus, the “water-in-salt” electrolyte plays a significant role in improving Zn^2+^ storage by suppressing the dissolution of vanadium-based cathodes.

Next, ex situ XRD was employed to provide further insight into the energy storage mechanism of the Zn/MnVOPO_4_·2H_2_O battery in the 21 m LiTFSI + 1 M Zn(CF_3_SO_3_)_2_ electrolyte (Figure 7g). Compared with the original sample of MnVOPO_4_·2H_2_O, the peak position of the (002) crystal plane in the initial state shifted from 12.8° to 13.67°, with slightly reduced layer spacing from 6.9 Å to 6.5 Å. This can be attributed to the interlayer water molecules exchanging with the water molecules in the electrolyte when the MnVOPO_4_·2H_2_O electrode came into contact with the aqueous electrolyte, resulting in a change in the number of interlayer water molecules in MnVOPO_4_·2H_2_O [37]. When discharging from a, at a fully charged state, to b, the peak slightly shifted to a lower angle, which indicates that the initially inserted Zn^2+^ ions widened the layer spacing. However, the repulsive force between layers was weakened after the increase in the number of inserted Zn^2+^ ions, which resulted in decreased layer spacing due to the strong electrostatic attraction interactions between the interlayer oxygen atoms and the inserted Zn^2+^ ions (from b to d). In the subsequent charging process, the change in the 2θ value of the (002) crystal surface was highly consistent with the reverse change in the discharge process, which indicates that the MnVOPO_4_·2H_2_O showed good cyclic reversibility in the 21 m LiTFSI + 1 M Zn(CF_3_SO_3_)_2_ electrolyte.

Moreover, ex situ XPS was used to explore the valence change in the active element during the (dis)charging process (Figure 7h). The peak position corresponding to V 2p in the fully discharged state shifted to a lower binding energy compared with the fully charged state, which indicates that the V ion gained electrons during the discharge process, and the valence decreased. Furthermore, electrons were lost in the charging process and increased the valence. The ex situ XPS results are highly consistent with the in situ XRD results, indicating that the reaction mechanism of this battery is the insertion/extraction reaction of Zn^2+^ in MnVOPO_4_·2H_2_O, accompanied by the oxidation/reduction of vanadium in the positive electrode material.

Hence, the electrochemical reaction of Zn/MnVOPO_4_·2H_2_O in the 21 m LiTFSI + 1 M Zn(CF_3_SO_3_)_2_ electrolyte is summarized as follows:

Cathode:

MnVOPO_4_·2H_2_O + xZn^2+^ + 2xe^−^ ↔ ZnxMnVOPO_4_·2H_2_O

Anode:

xZn ↔ xZn^2+^ + 2xe^−^

Total:

MnVOPO_4_·2H_2_O + xZn ↔ ZnxMnVOPO_4_·2H_2_O

## 4. Conclusions

In summary, the proposed “water-in-salt” electrolyte can effectively inhibit the decomposition of H_2_O and the dissolution of cathode materials by greatly reducing the content and reactivity of water. Moreover, the polyanion-based cathode has the advantage of a high voltage. Based on this, the MnVOPO_4_·nH_2_O//21 m LiTFSI + 1 M Zn(CF_3_SO_3_)_2_//Zn battery exhibited outstanding cycling performance. Its specific discharge capacity reached 152 mAh g^−^^1^ at a low current density of 0.2 A g^−^^1^ (approximately 130 mAh g^−^^1^ after 100 cycles). Furthermore, it achieved a high and stable discharge voltage of 1.39 V and a considerable energy density of 211.78 Wh kg^−^^1^. Finally, the energy storage mechanism of Zn^2+^ insertion/extraction was demonstrated using in situ XRD and in situ XPS technology. This strategy of using a “water-in-salt” electrolyte coupled with a high-voltage cathode could enhance the development of AZMBs and provide new insights for other types of metal-ion batteries.

## Figures and Tables

**Table 1:** The crystal face indexes, half-height widths, and grain sizes of the samples obtained using JADE 6.5.

Number	hkl	Distance in PDF Card	Distance	I % in PDF Card	I % in Result	D/nm
1	002	6.9034	6.9091	100	100	58.6
2	102	4.6125	4.6171	11	7	57.4
3	004	3.4535	3.4506	10	12.7	57.6
4	200	3.1013	3.1071	36	25.9	56.4
5	202	2.8285	2.8317	15	12.2	50.6

**Table 2:** Comparison of the current metrics with those reported in the literature.

Cathode	Discharge Medium Voltage	Ref.
MnVOPO_4_·2H_2_O	1.39 V	This work
K_0_._23_V_2_O_5_	Approximately 0.7 V	[28]
VS_2_	Approximately 0.65 V	[30]
VSe_2_	Approximately 0.75 V	[29]
PANI	Approximately 1.0 V	[32]
MoSe_2_	Approximately 0.3 V	[33]
VO_2_	Approximately 0.5 V	[34]
Na_3_V_2_(PO_4_)_3_	At 1.23 V	[35]
Na_3_V_2_(PO_4_)_3_	Approximately 1.1 V	[36]

## Data Availability

The original contributions presented in the study are included in the article, further inquiries can be directed to the corresponding author.
